# Supplementary material for: Prevalence of overweight and obesity among Kuwaiti adolescents and the perception of body weight by parents or friends
Source: PLoS One. 2022 Jan 4;17(1):e0262101. doi: 10.1371/journal.pone.0262101 (PMC8726464; doi:10.1371/journal.pone.0262101)
Supplement: Attachment — Submitted filename: Responses to the Reviewers Comments- Round -2 -8-10- 2021.pdf [file pone.0262101.s003.pdf]

Response to the Reviewers Comments (Round-2)

Revised MS title: Prevalence of overweight and obesity among Kuwaiti adolescents and the perception of body weight by parents or friends

ID #: (PONE-D-21-14400).

Submitted to: PLOS One

Dear respected Editor, PLOS One

Thank you again for your email regarding your request for a second revision of the above titled manuscript. Kindly, find below our itemized responses (point by point) to the comments made by the respected reviewer. All the changes made to the revised manuscript were included in a separate file labeled 'Revised Manuscript with Track Changes'. Also, an unmarked version of the revised paper without tracked changes (clean) was upload as a separate file labeled 'Manuscript'.

Reviewers' Comments	Authors' Reply
Reviewer #1: ABSTRACT<br>-The values in this sentence "...perceived their parents ($p < 0.001$) or friends ($p < 0.001$) as more likely to classify their weight as overweight or obese" (lines 52-53) refer to the obese individuals, not the overweight people (parents: $p = 0.011$ and friends: $p = 0.002$).	We modified the sentence to be as follow:<br>"… that adolescents perceived their parents ($p = 0.011$ and $p < 0.001$) or friends ($p = 0.002$ and $p < 0.001$) as more likely to classify their weight as overweight or obese, respectively.
MATERIALS AND METHODS<br>-Describe in the manuscript when the data collection was performed.	The data collection was performed during the Fall of 2019.
-"Chi-square tests of independence was used to examine the relationships between overweight or obesity category and selected variables related to the influence of media on adolescents' decision to lose weight or their perception of body thinness" (lines 164-166). It is not clear the sentence above on the use of variables related to the influence of media on adolescents' decision to lose weight or their perception of body thinness, because the researchers didn't show the results/discussed about it.	The statement was modified to the following:<br>Chi-square test of independence was used to examine the relationships of selected variables related to adolescents' perception of how parents' or friends' see their weight status relative to an adolescent's overweight or obesity versus non-overweight/non-obesity status.<br>The results are shown in table 3.

Furthermore, in lines 157-158 it is described that "In the present study, we used the data from part three only, which was related to body weight perception by parents or friends" and it is the part two that is related to "influence of media on dieting to lose weight". Explain.	It is correct that the data were from part three. However, we slightly modified the sentences to read as follow:<br>In the present study, we used the data from part three only, which was related to adolescent's body weight perception as seen by parents or friends.
RESULTS<br>-The 95% CI datum cited in the line 208 about overweight individuals is not compatible with the informed in the Table 5.	Thanks for the comments. We have corrected the wrong 95% CI datum shown in page 7, line 202.
DISCUSSION<br>-In the sentence "other studies published over the past 'seven' years [2-4]" (line 226) the reference of number 4 was published in 2004. Change the description of years.	We corrected the sentence so to read as follow:<br>"… other studies published over the past 15 years [2-4]."
-The researchers assessed the adolescents' perceptions of parents' and peers' opinions about their weight. I think it is valid to add other studies that have assessed the same (the adolescents' perceptions of how parents and peers assess their weight). And it would be interesting to describe the reason whereby you chose to collect this information from the adolescent, not by parents/peers.	Using PubMed and Google scholar search, we were unable to locate any study related to how parents (or friends) perceived adolescents' weight status as seen by the adolescents themselves. This is the major reason lead to the initiation of this study. It is also more important to assess the direct perception of the adolescents not the parents (or friend) about how they see other perception about their weight status.<br>We added a sentence in the strength of the present study indicating this aspect of the study.
Also, in the conclusion section the researchers mentioned of importance of parents' perception (and why not from friends?), lines 311-312.	Thanks. We added friends as well.
TABLE 1 - Wouldn't the variable "overweight or obesity status (%)" be "nutritional status"?	It can be both titles. However, we have chosen "overweight or obesity" as this is the stated definition in the extended International Obesity Task Force (IOTF) age- and sex-specific BMI cutoff reference standards [reference number 22].

-Please, review the manuscript writing, words as "Portuguese" (line 248), sentences (as in lines 270-273), among others.	Portuguese was corrected to Portugal.

End of report.

Hazzaa M. Al-Hazzaa, PhD, FACSM, FECSS
Lifestyle and Health Research Center, Health Science Research Center, Princess Nourah Bint Abdulrahman University, Riyadh, Saudi Arabia (halhazzaa@hotmail.com)
