# Supplementary material for: Prevalence of overweight and obesity among Kuwaiti adolescents and the perception of body weight by parents or friends
Source: PLoS One. 2022 Jan 4;17(1):e0262101. doi: 10.1371/journal.pone.0262101 (PMC8726464; doi:10.1371/journal.pone.0262101)
Supplement: Attachment — Submitted filename: Responses to the Reviewers Comments-15-8-2021.docx [file pone.0262101.s002.docx]

**Response to the Reviewers Comments**

**Revised MS title**: Prevalence of overweight and obesity among Kuwaiti adolescents and the perception of body weight by parents or friends (PONE-D-21-14400).

**Submitted to**: PLOS One

**________________________________________________________________________________**

Dear respected Editor, PLOS One

Thank you for your email regarding your request for a revision of the above titled manuscript. First, we would like to thank you and the reviewers for the constructive comments. Second, kindly, find below our itemized responses (point by point) to the comments made by the respected reviewers. All the changes made to the revised manuscript were included in a separate file labeled 'Revised Manuscript with Track Changes'. Also, an unmarked version of the revised paper without tracked changes (clean) was upload as a separate file labeled 'Manuscript'. Briefly, in the revised manuscript, we adopted all the comments raised by the reviewers.

| **Reviewers’ Comments** | **Authors’ Reply** |
| --- | --- |
| 1- **Title** I suggest that the title be Prevalence of overweight and obesity among Kuwaiti adolescents and the perception of body weight by parents or friends. | We modified the title according to the reviewer’s suggestion (title page in the revised manuscript with track changes). |
| 2. **Keywords**  I suggest replacing “weight estimation” and “obesity perception” with “body weight” and “weight perception”. | Done (page 2, lines 57 & 58) in the revised manuscript with track changes. |
| 3. **Introduction**  I suggest reversing the order of references 10 and 11 in the text (lines 85-90). | References 10 and 11 were reversed in the text and in the references section. Page 3, lines 85-87 in the revised manuscript with track changes. |
| 4. Present the abbreviation of the word “body mass index” (lines 83-84) instead of putting it into methods (line 132). | Thanks. Done. Page 3 line 83 in the revised manuscript with track changes. |
| 5. **Materials and Methods Study design and participants**  The researchers reported that they recruited “adolescents between the ages of 15 and 18 years” (lines 114-115). What was the criterion adopted for choosing this age group? | This is the age group in the secondary schools in Kuwait, as sample was selected from the secondary schools. |
| 6. What was the estimated sample size? Were there losses? | Regarding the estimated sample size: “A predetermined sample size of 368 participants for each sex group was calculated, so that the population proportion was assumed to be at 0.60 with a tolerated error of 0.05.” Page 4, lines 116-118 in the revised manuscript with track changes. |
| 7. How did the researchers assess whether the students suffered or not from diet-related disorders (line 122)? | Using questions inserted in the questionnaire. We included this statement in the participant section (page 4, line 126 in the revised manuscript with track changes). |
| 8. **Description of questionnaire**  The researchers did not described about the questions “Do your parents compare your weight with any of your brother/sister?” and “age at maturation”. How did you assess the maturation age? | We actually meant puberty, so we replaced the word “maturation” with the word “puberty” throughout the manuscript. Page 5, line 142 in the revised manuscript with track changes. |
| 9. **Statistical analysis**  In “Descriptive statistics were obtained for all variables...”, include the variables (line 157). | With all respect to the reviewer’s comment, there are so many variables and it is redundant to list all of them, as they are clearly presented in tables 1 to 5. |
| 10. In the sentence “Chi-square tests of proportions were used to test differences in selected variables related to the influence of media on adolescents’ decision to lose weight...”, explain this relation of media influence (lines 160-162). | We actually used the chi-square test of independence was used to examine the relationships between overweight or obesity category and selected variables related to the influence of media on adolescents’ decision to lose weight or their perception of body thinness.  And we modified the statement accordingly. Page 6, lines 164-168 in the revised manuscript with track changes. |
| 11. **Results**  The Table 3 refers to the weight status from individuals with “non-overweight or non-obesity” or “overweight or obesity”, thus why the title is written “relative to an adolescent’s overweight or obesity”? | We modified such statement to the following: “relative to an adolescent’s overweight or obesity versus non-overweight/non-obesity status”. Page 19, table 3, in the revised manuscript with track changes. |
| 12. **Discussion**  The researchers mentioned that “...higher rates among males in some countries and higher prevalence among females in other countries...”, I think it is interesting to include these countries (lines 238-241). | We added such information, so the statement is read now as “BMI centiles among 15-year old adolescents in an international sample showed statistically higher rates among males in some countries (United States: 13.9%, and Greek: 10.8%) and higher prevalence among females in other countries (United States: 15.1%, and Portuguese: 6.7%)”. Page 8, lines 245, 248 in the revised manuscript with track changes. |
| 13. The sentence mention about female adolescents and, what about female students? (lines 260-263). | We added the words “female”, so the sentences become “Korean male and female students”. Page 9, line 271 in the revised manuscript with track changes. |
| 14. Why did not the researchers assess the socioeconomic conditions, physical activity and food consumption? I think it is valid that you reassess and use these aspects as variables, due they are very important and can influence in the nutritional status of adolescents and in the weight perception. | When analyzing the data, we realized how important the socioeconomic status is. However, the study’s data were collected just before Covid-19 and it was difficult to go back and collect these parameters, as there were lockdown due to Covid-19. Page 2, line 61-62 in the clean revised manuscript. |
| 15. Include the contributions that this study give to the society. | We added the following statement at the end of the conclusion: “The present study updated the information related to the prevalence of overweight or obesity among Kuwaiti adolescents and showed the importance of parent’s perception of adolescent’s weight status.” Page 10, lines 310-312 in the revised manuscript with track changes. |
| **Journal (Editorial) requirements** | |
| 1. Please ensure that your manuscript meets PLOS ONE's style requirements, including those for file naming. | We modified the submitted files’ names. |
| 2. Please provide additional details regarding participant consent. In the ethics statement in the Methods and online submission information, please ensure that you have specified what type you obtained (for instance, written or verbal, and if verbal, how it was documented and witnessed). If your study included minors, state whether you obtained consent from parents or guardians. If the need for consent was waived by the ethics committee, please include this information. | We included the word “written” to the participant consent. Page 4, lines 128-129 in the revised manuscript with track changes. |
| 3. Thank you for stating the following in the Acknowledgments/Funding Section of your manuscript:  “Professor Hazzaa M. Al-Hazzaa’s research has been funded by the Deanship of Scientific Research at Princess Nourah bint Abdulrahman University through the Fast-track Research Funding Program.”  We note that you have provided funding information that is not currently declared in your Funding Statement. However, funding information should not appear in the Acknowledgments section or other areas of your manuscript. We will only publish funding information present in the Funding Statement section of the online submission form. | We removed the funding statement from the revised manuscript and added the funding information to the online submission form. Please see page 11, lines 323-326 in the revised manuscript with track changes. |
| Please remove any funding-related text from the manuscript and let us know how you would like to update your Funding Statement. Currently, your Funding Statement reads as follows: “The author(s) received no specific funding for this work.” | Removed. And funding statement was updated. |
| Please include your amended statements within your cover letter; we will change the online submission form on your behalf. | Included in the covering letter. |

End of report.

Hazzaa M. Al-Hazzaa, PhD, FACSM, FECSS

Lifestyle and Health Research Center, Health Science Research Center, Princess Nourah bint Abdulrahman University, Riyadh, Saudi Arabia ([halhazzaa@hotmail.com](mailto:halhazzaa@hotmail.com))
